# MiR-7 in Cancer Development

**DOI:** 10.3390/biomedicines9030325

**Published:** 2021-03-23

**Authors:** Petra Korać, Mariastefania Antica, Maja Matulić

**Affiliations:** 1 Department of Biology, Division of Molecular Biology, Faculty of Science, University of Zagreb, Horvatovac 102, 10000 Zagreb, Croatia; petra.korac@biol.pmf.hr; 2 Division of Molecular Biology, Rudjer Bosković Institute, Bijenička 54, 10000 Zagreb, Croatia; antica@irb.hr

**Keywords:** microRNAs, miR-7, gene expression, tumor suppressor, cancer cell

## Abstract

MicroRNAs (miRNAs) are short non-coding RNA involved in the regulation of specific mRNA translation. They participate in cellular signaling circuits and can act as oncogenes in tumor development, so-called oncomirs, as well as tumor suppressors. miR-7 is an ancient miRNA involved in the fine-tuning of several signaling pathways, acting mainly as tumor suppressor. Through downregulation of PI3K and MAPK pathways, its dominant role is the suppression of proliferation and survival, stimulation of apoptosis and inhibition of migration. Besides these functions, it has numerous additional roles in the differentiation process of different cell types, protection from stress and chromatin remodulation. One of the most investigated tissues is the brain, where its downregulation is linked with glioblastoma cell proliferation. Its deregulation is found also in other tumor types, such as in liver, lung and pancreas. In some types of lung and oral carcinoma, it can act as oncomir. miR-7 roles in cell fate determination and maintenance of cell homeostasis are still to be discovered, as well as the possibilities of its use as a specific biotherapeutic.

## 1. Introduction

MicroRNAs are short non-coding RNAs involved in the regulation of specific mRNA translation. Through this process, they regulate numerous cellular functions, participate in signaling circuits and fine-tune cellular differentiation. 

miRNAs (miRs) have a complex pathway of biogenesis and regulation of their function. While final miRNAs are short single-stranded noncoding RNAs of 20–23 nt, they start as pri-miRNAs, several hundred base pairs long with a complex formation pathway. These primary miRNAs are first processed by a microprocessor containing Drosha, an enzyme that cleaves the stem of a hairpin structure formed by future miR sequence and producing pre-miRNA. After nuclear export, further processing is done by Dicer in the cytoplasm, which removes the loop region and produces miRNA duplex. Only one strand of the duplex is chosen to become the mature miRNA, loaded on an RNA-induced silencing complex (RISC) containing the Argonaute protein. RISC complex with specific miR targets complementary mRNAs and fully complementary mRNA are degraded. Since mature miRNAs in higher eukaryotic cells most often are not fully complementary to their target mRNA, they can lead to translation inhibition [1].

Usually, one mRNA can be targeted by several miRNAs on its 3’UTR. It is supposed that the target site spacing can influence cooperative repression. Although a great number of genes can be influenced by a single miR, in general, miRs act according to the cellular program in a specific cell type and target only a subset of transcripts [2]. However, the regulation of these processes is still poorly understood.

One of the first known, and also most investigated miRNAs is miR-7. The seed sequence GGAAGA is evolutionarily conserved and is found in Nematodes, Insects and Vertebrates [3]. In Mammals miR-7 dominantly acts as a tumor suppressor and regulates several basic cellular processes, which include proliferation, differentiation, apoptosis, migration and expression of stem cell features. It was also one of the miRs used for the classification of the regulatory clusters. Most discoveries are in regard to its role in the brain and sensory cell differentiation in man and Drosophila, respectively. Li X, and his collaborators (2009) suggest that miRs, in general, may stabilize different regulatory networks depending on the conditions of environmental fluctuation during development [4]. This hypothesis was developed on an example of miR-7, participating in Notch and Epidermal growth factor receptor (EGFR) coherent and incoherent feedforward loops during photoreceptor determination in *Drosophila*. On the other hand, miR-7 downregulation is linked to cell proliferation in many tumors, and its regulation is tightly connected with differentiation processes in the pancreas, brain and other organs [5,6].

miR-7 is encoded in three different sites in the human genome. *MIR7-1* sequence is present inside the last intron of the heterogeneous nuclear ribonucleoprotein K (hnRNPK) gene, on chromosome 9 (9q21.32) and is considered to be the dominant gene responsible for miR-7 expression. *MIR7-2* sequence is present in the intergenic region on chromosome 15, and *MIR7-3* in the intron of pituitary gland specific factor 1 gene (*PGSF1*) or *MIR-7* host gene on chromosome 19 [7].

## 2. Regulation of MiR-7 Expression

miRNA genes, like the protein-coding genes, have a regulated promoter and their products are members of signaling circuits of different cellular processes. miRs are also regulated at several steps during processing into their active form by means of binding to different proteins [8]. miRs can bind different long non-coding RNAs and circular RNAs either to be degraded or to be “preserved” for later function. Different RNA classes can function as miR “sponges” and bind miRs to keep them out of function: 3’UTR mRNA [9], long non-coding RNAs (lncRNAs) and circular RNAs. Different proteins can also regulate pri-miR degradation [1].

miR-7 is considered to be a network stabilizer, connecting different signaling pathways through feedback and feedforward loops [4]. Its function in buffering gene expression and providing robustness in cell response was demonstrated. Caygill and Brand (2017) showed on the *Drosophila* model where miR-7 targeted the Notch pathway, that miR-7 buffers the differentiation of the neuroepithelial cells into neuroblasts. Its role was to enable precision in the process despite conditions of environmental stress [10].

As a tumor suppressor, miR-7 expression is often downregulated in different cancer cells (i.e., in brain, lung and colon cancer cells [11,12,13]). Interestingly, it is also involved in signaling circuits directing differentiation in different tissues and it is regulated by specific transcription factors [6,14,15,16]. miR-7 promoter was found to be silenced by DNA methylation in cancer stem cells [17]. In breast carcinoma, its expression is estrogen-dependent [18]. Duex et al. found miR-7 to be in a signaling loop with EGFR through Usp18 (Ubp43), a ubiquitin-specific peptidase, whose downregulation elevates miR-7 levels [19]. It was also found that Hepatitis B virus (HBV) protein HBx can upregulate miR-7 expression through EGFR [20] and in breast cells by hepatocyte growth factor (HGF) [21]. However, miR-7 inhibition promotes breast cancer metastasis [22].

miR-7 was found to belong to a p53-dependent non-coding RNA network [23,24], as well as the Myc signaling circuit [25]. Hansen et al. described the existence of circular RNAs, which can pair with complementary miRNAs [26]. Circular RNAs have a structure of covalently closed single-stranded RNA molecules, produced by a specific type of splicing. These molecules are more stable than linear. Some of them can act as miR sponges: RNA molecules, which contain multiple target sites complementary to a specific miR and influence its activities by binding to it. The first such molecule was detected in neurons and it was Cdr1as (ciRS-7) regulating miR-7. It contained miR-7 sequences transcribed in the antisense orientation from the *CDR1* gene, forming circular RNA (circRNA) Cdr1as with more than 70 binding sites for miR-7 and one perfectly complementary site for miR-671 [26,27,28]. It seems that Cdr1as binds miR-7s and serves as their reservoir, and their release is regulated with miR-671, which causes cleavage of Cdr1as and liberation of miR-7s to exert their activities. Furthermore, Kleaveland et al. ( found miR-7 to be a member of a regulatory network consisting of four ncRNAs: one long ncRNA, one circular and two microRNAs, in the mouse cerebellum [29]. Cyrano is a long ncRNA, which pairs to miR-7 and triggers its destruction. At the same time, this long ncRNA enables upregulation of circular Cdr1as, otherwise downregulated by miR-7. miR-671 was found to be involved in Cdr1as destruction.

Numerous long noncoding RNAs were found to bind to miR-7 and downregulate its activities: LINC00115 and XIST in breast cancer [22,30], LINC00240 in lung cancer [31], RSC1-AS1 in hepatocellular carcinoma, TINCR and Zing Finger Antisense 1 (ZFAS1) in breast and colorectal cancer [32,33,34], LPP-AS2 in glioma cells [35], etc. LncRNA SOX21-AS1 influenced cervical cancer progression by inhibiting miR-7/VDAC1 (voltage-dependent anion channel 1) [36]. lncRNA KCNQ1OT1 modulated cell resistance to chemotherapy [37], and lncRNA FOXD2-AS1 was found to bind miR-7 in thyroid cancer, upregulating the expression of hTERT [38]. lncRNA UCA1 downregulated miR-7, influencing the EGFR axis in gastric cancer cells resistant to hypoxia [39]. Upregulation of long noncoding RNA ANRIL caused by hypoxia modulated miR-7/SIRT1 axis and protected cells from cell death [40]. lncRNA CASC21 influenced miR-7/YAP1 signaling in colorectal cancer [41], and lncRNA Oip5-as1 in stem cells was found to modulate NANOG expression [42].

Several circular RNAs, besides ciRS, also regulate miR-7 and its downstream targets: circHIPK3 in colorectal cancer, circ-0015756 in hepatocellular carcinoma influencing downstream FAK [43,44], hsa_circRNA_0006528 in breast cancer influencing proliferation through MAPK/ERK pathway [45], circ-U2AF1 in gliomas influencing the expression of NOVA 2 [46], circ-TFCP2L1 decreasing mir-7-PAK1 signaling [47], circAkap17b regulating FSH secretion in pituitary gland [48]. circSNCA, *SNCA* and miR-7 were found to be regulated by endogenous competition and could influence the progression of Parkinson’s disease [49].

Other types of RNA can also modulate miR-7 activity: 3′UTR Ube3a-1 mRNA [9] and Small Nucleolar RNA Host Gene 15 (SNHG15) regulating Klf4 through miR-7 [50].

There are also several proteins, which influence miR-7 maturation. Wang et al. described miR-7 regulation by protein quaking isoforms (QKI) [51]. The QKI proteins have heteronuclear ribonucleoprotein particle K (hnRNPK) homology KH and belong to RNA binding proteins. These proteins interact with a QKI response element sequence in introns and mature mRNAs, and it was shown that nuclear isoforms QKI-5 and QKI-6 associated with pri-miR-7-1 to prevent its processing. They were also found to interact with Ago2, during stress conditions. Similarly, it was shown that miR-7 can be downregulated by NF90-NF45 complex, through the binding of this double-stranded RNA-binding protein complex to primary miR-7 [52]. miR-7, in turn, targeted the coding region of NF90 mRNA. Nerve cells have a posttranscriptional regulation of miR-7 through the expression of Musashi homolog 2 (MSI2) and Hu antigen R (HuR), miR processing inhibitors and tissue-specific factors, regulating miR-7 expression and activity during neural differentiation [8]. A similar regulation was found in human lung cancer cells as a response to TLR9 signaling [53]. In addition, mechanisms of miR-7 targeted degradation linked with its 3′ end modifications were recently discovered [54].

**Figure 1:**
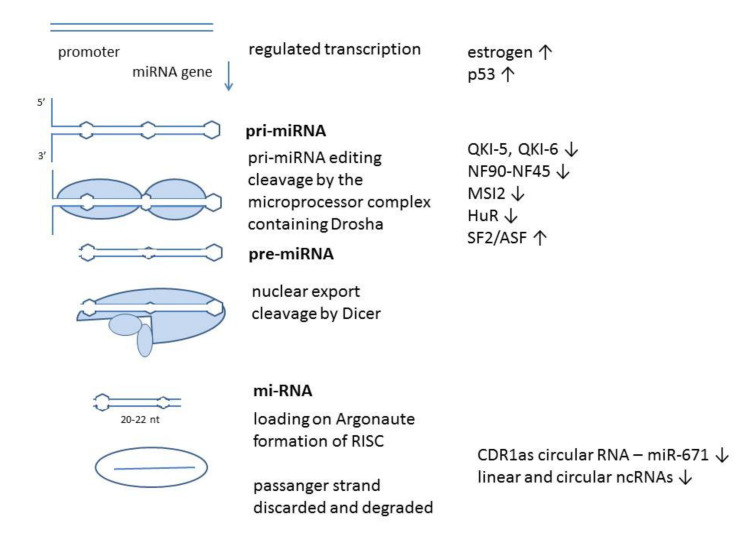
Biogenesis of miRNA. miRNA are transcribed from their genes regulated by promoters. Primary or pri-miRNA is several hundred base pairs long and has a form of a hairpin. It is processed by a microprocessor, a complex containing Drosha enzyme which removes the stem of the structure. Such pre-miRNA is exported from the nucleus and further cleaved by Dicer. miRNA duplex of 20–22 22 nt is produced. One strand of the duplex becomes the mature miRNA loaded on RNA-induced silencing complex (RISC), containing Argonaute protein. miRNA targets mRNA complementary to its sequence and directs it to degradation or inhibits translation, depending on the level of complementarity. Some of the known signaling molecules regulating miR-7 expression are shown [1,8,18,23,26,31,32,33,34,35,36,37,38,39,40,41,42,43,44,45,46,47,48,49,50,51,55].

On the other side, SF2/ASF increases Drosha cleavage of primary miR-7 transcript and promotes miR-7 maturation, and miR-7 in the feedback loop can decrease SF2/ASF expression. This molecule does not only affect miR-7, but also other miRs, coordinating their splicing regulation and gene repression [55] (Figure 1).

## 3. MiR-7 and Chromatin Regulation

miR-7 was found to regulate a number of genes involved in chromatin modulation. It can downregulate histone methyl-transferase gene, *SETDB1* in different types of cancer cells [56,57], as well as *TET2* and *SMARCD1* [58,59]. It can also influence global cellular expression through the regulation of master transcription factors, such as *KLF4*, and thus impact the fate of cancer stem cells and human embryonic stem cells [60]. miR-7 is also found in extracellular vesicles and besides the possibility to influence the fate of the cell where it is expressed, it could also interfere with the biology of the cells to which it is delivered [61].

## 4. MiR-7 in Nerve Cells and Glioblastoma

**Figure 2:**
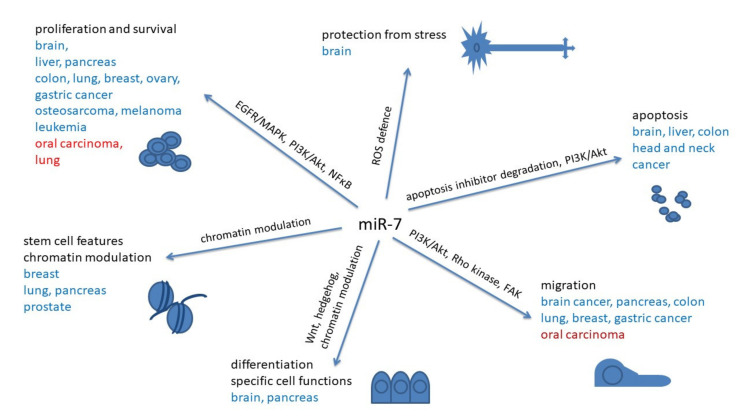
Effects of miR-7 on the process of carcinogenesis in different types of cancer. blue: tumor suppressor’s activities; red: activities as oncomirs.

In brain development, a fine regulation of cell proliferation, cell differentiation and regulation of symmetric and asymmetric division, as well as cell migration is necessary. It seems that miR-7 has a role in fine-tuning of these processes, in general as a suppressor of proliferation (Table 1, Table 2, Table 3 and Table 4, Figure 2).

It has spatiotemporal-dependent expression and regulation [28], and it is found in discrete brain regions [3]. It can also have specific subcellular localization, different in the cell body and neurites. One example is miR-7 role in dopaminergic neuron differentiation by fine-tuning *Pax6* expression [5]. miR-7 also regulates other neural fate markers, elements of the Wnt pathway, interferes with Hedgehog and Notch signaling and takes part in the differentiation process [134]. miR-7 regulates both, specific nerve functions (such as synaptic [144]) and master regulators (such as HoxD family members). It is detected as one of the miRNAs forming “miR signature” in neural stem and neural cancer stem cells [154], which is in accordance with its role in differentiation and proliferation. Interestingly, miR-7 is 40 times more abundant in neurons than in astrocytes (Table 1).

Besides influencing cell differentiation, and thus indirectly interfering with it, miR-7 can also directly inhibit cell proliferation (Figure 2). In glioblastoma and neuroblastoma miR-7 was found to be downregulated compared to normal tissue, indicating its role as a tumor suppressor [11,62]. The functions of miR-7 in glioblastomas are mainly linked to its influence on cell proliferation, differentiation, apoptosis and migration. Although some glioblastoma cells can be refractory to miR-7 expression, its downregulation is often found in nerve cell tumors. Saydam et al. found its downregulation to be the typical miR schwannoma characteristic signature [11].

One of the first detected and most investigated targets of miR-7 is Epidermal growth factor receptor, EGFR, whose protein expression is decreased by miR activity. EGFR is linked to several important proliferation-inducing pathways, such as PI3K/Akt and MAPK and their downregulation leads to decreased activation of the Akt and ERK1/2. Kefas found that miR-7 directly regulates EGFR expression [62].

miR-7 targets are also several other proteins involved in downstream signaling. In the PI3K/Akt pathway, these are Akt pathway regulators IRS-1 and IRS-2, PI3K subunits (PIK3R3 and PIK3CD), mTOR [66], and PAK1 (p21/Cdc42/Rac1-activated kinase) [67,155]. The latter is potentially involved not only in oncogenic signaling through EGFR/Akt, but also in motility, regulation of cytoskeleton and apoptosis [63]. On the MAPK pathway, miR-7 influences Raf1 and ARF4 (ADP-ribosylation factor 4) expression, which modulates activation of phospholipase D2 (PLD2) and downstream activation of AP-1 [67,155]. Webster et al. found its influence on JNK and CAMK pathways [63]. In addition, Duex et al. found miR-7 to be involved in the signaling loop with EGFR through Usp18 (Ubp43), a ubiquitin-specific peptidase, whose downregulation elevates miR-7 levels [19].

miR-7 is also involved in the regulation of cell survival [67,84] as it downregulates pro-survival proteins IRS-1, IGF-1R, PAK1, and Raf-1 and leads to the reduction in cell viability. Zhang X et al. found that the expression pattern of miR-7 correlates with the glioblastoma cells’ sensitivity to apoptosis induced by TRAIL, a TNF family member [110]. XIAP, an apoptosis inhibitor, was detected as a direct miR-7 target (Table 1, Table 2 and Table 3).

In another experimental setting, Kabaria et al. found that miR-7 targeted 3’UTR of Keap1 in human neuroblastoma cells [119]. Keap1 takes part in the regulation of Nrf2, a transcription factor involved in the expression of many antioxidant and detoxifying genes in reactive oxygen species (ROS) defense. miR-7, therefore, participated in cellular protection from oxidative stress. In neuroblastoma cells, Sirtuin (Sirt 1) was found to be a direct target of miR-7, and a link to the regulation of oxygen-glucose deprivation and cerebral injury [116]. It was found that miR-7 can target VDAC1, voltage-dependent anion channel, a part of the mitochondrial permeability transition pore, leading to the decrease in the intracellular ROS and protection against mitochondrial dysfunction and cytotoxicity [117]. Jia et al. compared RNA expression in glioblastoma cell lines differently sensitive on alkylation DNA damage and found miR-7 to be downregulated in the resistant cells [115]. They showed that miR-7 upregulation increased the cell sensitivity to alkylation. As a direct target, transcription factor YY1 was identified. However, it is also possible that in glioblastoma cell lines cell-specific regulation exists and that not all cell lines are responsive to miR-7 expression [8,156].

miR-7 also targets the expression of proteins involved in migration and metastasis [99]. Increased expression of miR-7 inhibited migration and invasion through downregulation of MMP-2, MMP-9 and FAK, a kinase involved in motility. Different targets were found to link miR-7 to actin cytoskeleton: Rho GTPases, Ack1 and PAK. In addition, in glioblastoma its target was a special AT-rich sequence binding protein 1 (SATB1), a protein able to promote migration and invasion [105]. 

Pan CM et al. found miR-7 to target TBX2 mRNA, and due to miR-7 downregulation in glioblastomas, TBX2 is increased [96]. Its high expression correlated with poor prognosis and higher invasivity of glioblastoma cells, EMT features and pulmonary metastasis. TBX2 is involved in the developmental processes and morphogenesis of different organs. It represses E-cadherin and increases the invasiveness of breast cancer cells. miR-7 also influenced TFF3, a signaling molecule downstream of PI3K/Akt pathway. Its downregulation decreased migration and invasion. This process can be reversed by a glioblastoma cell treatment with a glycolytic inhibitor which reduces the expression of miR-7 [97].

## 5. MiR-7 Role in Gastrointestinal Tumours

In gastric cancer (GC) patients, miR-7 deregulation consequently leads to increased cell proliferation, tumorigenesis and poor survival. In gastric cancer cells, besides targeting the EGFR pathway, miR-7 targets the IGF1R and downstream RELA and FOS [77]. miR-7 indirectly influences RELA activation, through targeting IKKeta. Through the feedback circuit, the NF-kappaB pathway regulates the miR-7 expression. In addition, miR-7 can downregulate the IGF1R-Snail pathway, which is involved in epithelial-mesenchymal transition [74,77]. Similar pathways were influenced in tongue squamous cell carcinoma [71]. Recently it was found that miR-7 could target lactate dehydrogenase A (LDH-A) in gastric cancer cells, so its downregulation can influence glycolysis, cell proliferation and sensitivity to chemotherapy [150].

In oral squamous cell carcinoma cells miR-7 regulated the expression of RECK, which acts as a metalloproteinase inhibitor and can suppress cell proliferation and migration. Therefore, miR-7 acted as an oncogene, and RECK inhibition was associated with poor prognosis and aggressiveness of tumors [87] (Figure 2).

miR-7 has also been reported to target a specific set of genes in the liver. Some of them code for proteins involved in cell cycle and apoptosis regulation, such as CCNE1 [83], Bcl-2 and XIAP. In hepatocellular carcinoma cells miR-7 directly regulates *CUL5*, influencing cell proliferation and inducing cell cycle arrest [92]. As miR-7 targets Notch3, its downregulation leads to Notch signaling activation in the same type of cancer cells [157]. Besides Notch3, Notch4 and VE cadherin were also found to be miR-7 targets [107]. miR-7 also downregulates *VDAC1* in hepatocellular carcinoma and influences proliferation and migration [118], as well as the fibroblast growth factor receptor FGFR4, a key molecule for liver protection from chronic injury. In the conditions of increased fibrosis miR-7 was found to be upregulated and promoted HSC proliferation and activation as a consequence of *FGFR4* downregulation [132].

miR-7 is involved in the differentiation of pancreatic endocrine cells [6]. In pancreatic carcinoma, miR-7 can suppress NFAT. This transcription factor can regulate epithelial-mesenchymal transition and act as an oncogene in pancreatic carcinoma cells [106]. Downing et al. found miR-7 to directly target *REG1*, a protein that increases proliferation and influences apoptosis and differentiation of pancreatic cells [124]. miR-7 was found to suppress SOX18 and to influence the gp130/JAK2/STAT3 pathway. Wang et al. found miR-7 to target members of the mTOR signaling pathway (p70S6K, eIF4E, Mapkap1, Mknk1 and MknK2) [75], influencing cell proliferation, as well as MAP3K9 [76]. In addition, miR-7 targets also SET8, a histone methyltransferase, thus potentially influencing the expression of a number of downstream genes [56] (Table 4).

In colon cancer cells miR-7 also suppresses proliferation, increases apoptosis and causes cell-cycle arrest, by targeting YY1 and by influencing downstream p53, caspases and c-jun, as well as wnt signaling (through beta-catenin, survivin and FGF4) [13]. Neil, an endonuclease that inhibits apoptosis and increases cell survival and proliferation was found to be regulated by miR7 [113].

Other targets are *TYRO3*, influencing PI3K/Akt/mTOR pathway [93], *TRIP6* which regulates proliferation and metastases [90], *FAK* [101] and *XRCC2*, a gene involved in homologous recombination repair pathway [123].

## 6. MiR-7 Roles in Lung Cancer

Promoter mutation of miR-7 was found to be associated with a poor prognosis of lung cancer [12]. The main targets released from miR-7 downregulation are those of EGFR and PIK3/Akt pathways, apoptosis inhibitors [109], and proteins involved in migration, FAK, PAK2 and NOVA2 [12,72,94,102,103]. PIK3/Akt signaling also connects TLR9 and miR-7 regulation [65]. However, Chou found that miR-7 could act as an oncomir in lung tumorigenesis [88]. EGFR, through the Ras/ERK/Myc pathway, increased the production of miR-7-1, which targets ERF, a transcriptional repressor. Therefore, in carcinoma samples, a positive correlation between EGFR and miR-7 expression was found, and miR-7 increased cell proliferation and tumor volume. Another example of oncogene activity was miR-7 modulation of the MYC pathway, in a positive feedback loop. The miRNA target is *AMBRA1*, an important regulator of early autophagy and a mediator in MYC dephosphorylation [158].

Hong et al. identified *SMARCD1*, a chromatin remodeling protein, to be a direct target of miR-7 in lung cancer cells [59]. They concluded that miR-7 influences the coupling of SMARCD1 with p53, which leads to an increased chemoresistance of lung cancer cells. miR-7 also downregulates PARP1, thus influencing DNA homologous recombination repair and survival after Adriamycin treatment of small cell lung cancer cells [121]. Furthermore, miR-7 modulates chemoresistance by targeting the multidrug resistance-associated protein MRP1/ABCC1 [125].

In addition to that, miR-7 was found to target several proteins linked to protein degradation as PA28gamma, a proteasome activator, targeted in non-small cell lung carcinoma [140]. O-GlcNAcyl Transferase (OGT), an enzyme involved in O-linked *N*-acetylglucosaminylation and contributing to cancer phenotype, is regulated by miR-7 [147]. In nasopharyngeal carcinoma cells, miR-7 was found to regulate the expression of enolase, ENO2, and therefore its downregulation can influence cell glycolysis [149].

## 7. MiR-7 Roles in Melanoma and Skin Cancer

Similarly to its role in other tissues, in melanoma cells, miR-7 takes part in the suppression of proliferation. However, as melanoma cells are not typically EGFR-driven, Giles et al. found miR-7 to target RelA and thus inhibit NF-κB activity and its downstream genes, such as *IL-1β*, *IL-6* and *IL-8* [70]. The analysis of melanoma patient samples revealed a correlation between RelA expression and poor survival.

On the contrary, Meza-Sosa et al. found *KLF4* to be a miR-7 direct target in epithelial cells, and miR-7 overexpression in lung and skin epithelial cells enhanced cell proliferation, migration and tumorigenesis [84]. Tumors with an increased miR-7 had a decreased p21 and cyclin D. In thyroid papillary cancer, miR-7 targeted *CKS2*, a cyclin-dependent kinase regulator, and downstream cyclin B1 and cdk1 [89]. As a target, also *PAK1* was detected [95].

It has been shown that in the cancer-associated fibroblasts of head and neck cancers, overexpression of miR-7 downregulates *RSSF2*, a proapoptotic molecule influencing proliferation and migration, and decreases the secretion of a tumor suppressor PAR-4 (prostate apoptosis response 4) [159]. In the human ocular tissue, miR-7 targets *RGS5*, a regulator of G protein signaling [139].

## 8. MiR-7 Roles in Breast, Prostate and Ovarian Cancer

In breast cancer miR-7 inhibits the metastases and influences epithelial-mesenchymal transition by targeting FAK, a kinase that acts as a mediator in ECM-integrin signaling [100]. Overexpression of miR-7 induces an increase in E cadherin and downregulation of mesenchymal proteins, suppresses proliferation, anchorage-independent growth, migration and invasion, as well as anchorage-independent growth in matrigel. The level of miR-7 is associated with the aggressiveness of estrogen receptor-positive breast tumors [160]. It also targets proteasome activator subunit 3 (*REGγ*) and contributes to the decrease in the cancer stem cell population survival, proliferation and migration [30,91,112,161]. Several miR-7 targets influence chemotherapy resistance, such as members of EGFR/PI3K signaling, *BRCA1, LASP1, BCL-2* and *MRP1* [104,162,163]. Okuda et al. (2013) found that miR-7 suppresses the ability of breast cancer stem cells to metastasize to the brain [85]. The correlation was found with miR-7 modulation of KLF4 expression, involved in stem cell biology. In addition, in a breast cancer cell line miR-7 was also found to be in regulation circuit with HOXD10, and, together with miR-218, to downregulate HoxB3 [69,120]. These changes were further connected with increased activity of other tumor suppressors, RASSF1A and Claudin-6 through epigenetic regulation, leading to cell cycle inhibition. Seong et al. found miR-7 to target *REDD1,* a negative regulator of mTOR signaling in the stress conditions [122]. miR-7 was therefore assigned to so-called hypoxamirs, miRNAs involved in hypoxic response. In HeLa cells, hypoxia caused downregulation of miR-7, in order to increase REDD1 level and inhibit mTOR signaling. In prostate cancer, it was found that miR-7 can regulate the expression of AXL, a receptor tyrosine kinase, associated with tumorigenesis, inhibition of apoptosis and EMT, often deregulated in different types of carcinomas [164]. miR-7 also inhibited the stemness of prostate stem cancer cells through repression of *KLF4* and PI3K/Akt/p21 downstream pathway [165].

miR-7 overexpression in hamster ovary cell line CHO decreased the cell proliferation, without influencing viability. Transient transfection of CHO led to upregulation of nearly 200 genes and downregulation of around 350 genes. The pathways involved included translation, RNA and DNA processing, secretion and protein folding. miR-7 has been found to target regulators of G1-S transition, *Skp2* and *Psme*, to upregulate p27KIP and arrest the cells in the G1 phase. Furthermore, it was found that miR-7 coordinately changes the levels of many genes in order to maintain homeostasis under the arrest conditions. It regulates *Rad54L*, a DNA repair protein, and influences the proapoptotic regulator p53 and the antiapoptotic Akt pathway to insure cell survival [82,166].

In testicular germ cell tumors, miR-7 was found to be one of the four hub miRNAs in regulatory networks of nonseminoma tumors [167].

In addition, expression of miR-7 was found increased in renal cell carcinomas in comparison with normal tissue, suggesting its activities as an oncogene [168].

## 9. MiR-7 Roles in Mesenchymal Tissue and Tumours

In osteosarcoma miR-7 influences *IGF1R*, and in paediatric rhabdomyosarcomas targets *SLC25A37* and *TIMM50*, two mitochondrial proteins, important for the induction of cell death [79,111]. In osteosarcomas, miR-7 is supposed to be a regulating link between Linc00852 lncRNA, and AXL, a tyrosine kinase involved in tumor growth [169].

## 10. MiR-7 Roles in Leukaemia

miR-7 had a low expression in haematopoietic cells and in B-chronic lymphocytic leukaemia (Antica et al. unpublished results). In chronic myeloid leukaemia, it was found to interfere with Bcr/Abl signaling [170]. A higher expression of miR-7 was found in acute lymphocytic leukaemia (ALL) patients with CNS relapse compared to those without [171]. In B cell lymphoma it was found to be regulated by c-Myc [172]. In T-cell acute lymphocytic leukaemia (T-ALL), upregulation of long noncoding RNA ANRIL caused miR-7 sponging, binding multiple tandem miRNAs through response elements binding seed sequences, in order to sequester them from their target sequences. Consequently, *TCF4*, a miR-7 direct target, is upregulated and is involved in the disease progression [173]. In T-ALL, miR-7 was found to bind to *TAL1*, coding for T-cell acute lymphocytic leukaemia protein. In T-ALL, expression of miR-7 is often attenuated, while TAL1 expression is increased and solicitates cell proliferation [80]. In Non-Hodgkin lymphoma cells, miR-7 regulates migration and chemoresistance through KLF4 and YY1 [108] and miR-7 downregulation can increase the aggressiveness of follicular lymphoma by FasL upregulation in macrophages which modulate immunosuppressive stroma [174].

## 11. Conclusions

miR-7 is one of the most conserved and oldest miRs, and is engaged in numerous signaling circuits involved in differentiation, regulation of proliferation, apoptosis and migration. It targets numerous mRNAs depending on the intracellular milieu and is also regulated by different transcription factors and molecules involved in its processing and degradation. It was suggested that its role could be to buffer cellular processes under stress conditions and to coordinate cell proliferation with other functions. This could be the reason for its involvement in numerous diseases. In most tumors its expression is downregulated, as its dominant activity is tumor suppression by inhibition of cell proliferation and survival. In some cancer types, it acts as an oncomir, stressing the importance of nuances of signaling circuits in which it is involved. We believe that numerous functions in the maintenance of cell homeostasis and cell fate determination are still to be discovered. 

## Figures and Tables

**Table 1:** miR-7 target genes/proteins in proliferation.

Gene/Protein	Cell Type	Pathway	Function	Reference
EGFR	glioblastoma schwanoma lung cancer melanoma	EGFR signaling	inhibition of proliferation	[11,12,62,63,64]
PIK3R3 PIK3CD PI3K	glioblastoma lung cancer	PI3K/Akt pathway TLR9 pathway	proliferation inhibition	[65,66,67,68]
IRS-1, IRS-2	glioblastoma tong squamous cell carcinoma melanoma	PI3K/Akt pathway	inhibition of proliferation viability	[62,69,70,71]
Raf1	glioblastoma	EGFR signaling	inhibition of proliferation	[63,67,72,73]
FOS	gastric cancer	MAPK signaling	inhibition of proliferation	[74]
ARF4	glioblastoma	MAPK signaling	inhibition of proliferation	[63]
mTOR p70S6K eIF4E Mapkap1 Mknk1 Mknk2	glioblastoma pancreas	PI3K/Akt pathway	proliferation inhibition	[66,75]
MAP3K9	pancreatic cancer	MAPK pathway	inhibition of proliferation and migration	[76]
IGF-1R	gastric metastasis glioma tong squamous carcinoma osteosarcoma	PI3K/Akt pathway IGF1-Snail EMT	inhibition of migration and survival	[71,77,78,79]
TAL1	T acute lymphoblastic leukaemia	proliferation	inhibition of proliferation	[80]
RELA	gastric cancer melanoma	NFkappa B signaling	inhibition of proliferation	[74,81]
IKK eta	gastric cancer	NF kappa B	regulation of proliferation	[74]
Skp2 Psme	ovary cells	cell cycle regulation	cell cycle arrest	[82]
CCNE	liver hepatocellular carcinoma	cell cycle	inhibition of proliferation	[83]
KLF4	epithelial brain metastasis endothelial cells lung	stem cell regulation	proliferation migration angiogenesis	[84,85,86]
RECK reversion inducing cysteine-rich protein with kazal motifs	oral cancer	metalloproteinase inhibitor	increase in proliferation	[87]
ERF Ets2 transcriptional repressor	lung cancer	MAPK pathway	increase in proliferation	[88]
CKS2 cyclin-dependent kinase regulatory subunit 2	thyroid cancer	cell cycle	inhibition of proliferation	[89]
TRIP6 thyroid receptor interactor protein	colorectal cancer	proliferation	inhibition of proliferation and metastasis	[90]
ALDJ1A3	breast cancer	stem cell survival	decrease in stem cell survival	[91]
CUL5 cullin5	hepatocellular	ubiquitination and protein degradation	inhibition of proliferation cell cycle arrest	[92]
TYRO3	colorectal cancer	PI3K/Akt/mTOR	inhibition of proliferation	[93]

**Table 2:** miR-7 target genes/proteins involved in migration.

Gene/Protein	Cell Type	Pathway	Function	Reference
PAK2 PAK1	lung carcinoma thyroid cancer gliomas tong squamous cell carcinomaschwanoma	Rho kinase effector	inhibition of proliferation, motility, regulation of cytoskeleton apoptosis	[11,63,67,69,71,94,95]
TBX2 T-Box2	glioblastoma	differentiation, EMT	inhibition of invasiveness	[96]
trefoil factor 3	glioblastoma	PI3K/Akt pathway	inhibition of migration	[97]
cdc42	brain damaged	Rho kinase	inhibition of migration and proliferation	[98]
Ack1 associated cdc42 kinase 1	schwannoma	Rho pathways citoskeleton regulation	inhibition of migration	[11]
FAK FAK (PTK2)	glioblastoma breast cancer colon cancer lung cancer	citoskeleton regulation	inhibition of migration and proliferation	[99,100,101,102]
NOVA2	lung carcinoma		inhibition of migration	[103]
LASP1	breast cancer			[104]
SATB1 special AT rich sequence binding protein	glioblastoma		inhibition of migration and invasion	[105]
Slug	breast cancer	EMT	decrease in migration	[30]
NFAT	pancreas	EMT	inhibition of migration	[106]
VE cadherin Notch4	hepatocellular carcinoma		inhibition of migration	[107]
KLF4 YY1	Non-Hodgin lymphoma		inhibition of migration and chemosensitivity	[108]

**Table 3:** miR-7 target genes/proteins involved in apoptosis and protection from stress.

Gene/Protein	Cell Type	Pathway	Function	Reference
BCL-2	lung, liver	apoptosis	apoptosis	[109]
XIAP	glioblastoma cervical cancer hepatocellular carcinoma lung	apoptosis	apoptosis	[68,72,110]
SLC25A37 TIMM50	rhabdomyosarcoma	mitochondria	induction of cell death ??	[111]
REGγ proteasome activator subunit	breast cancer	proteasome	inhibition of proliferation increase of apoptosis	[112]
NEIL Nei endonuclease VIII-like 1	colorectal cancer	inhibition of apoptosis, proliferation	inhibition of proliferation and survival	[113]
UBE2A	brain	ubiquitination and protein degradation	amyloid peptide proteolysis	[114]
YY1	glioblastoma colon cancer	p53 pathway cell cycle arrest wnt signaling	resistance to alkylation	[13,115]
1BRCA1	breast cancer	DNA repair	decrease in survival	[104]
Sirtuin /Sirt1	neuroblastoma	regulation of oxygen-glucose deprivation	protection from damage	[116]
VDAC	neuroblastoma hepatocellular carcinoma	ion channel on mitochondria; ROS defense	protection from oxidative stress	[117,118]
KEAP1	neuroblastoma	ROS defense	protection from oxidative stress	[119]
HOXB3	breast cancer retinal epithelial cells	glucose metabolism PI3K/Akt/mTOR	reduction of high glucose damage	[120]
PARP1	lung cancer cells	DNA repair	decreased DNA repair and survival	[121]
REDD1 regulated in development and DNA damage response 1	cervical carcinoma cells under hypoxia	DNA damage response	hypoxamir proliferation modulation	[122]
SMARCD1	lung cancer cells	chromatin regulator p53 pathway	increased chemoresistance	[59]
XRCC2	colorectal cancer cells	DNA repair	proliferation inhibition, induction of apoptosis	[123]
Rad54L	ovary cells	DNA damage repair	survival under cell cycle arrest conditions	[82]
REG1 regenerating islet-derived protein	pancreas	response to glucose starvation	inhibition of proliferation, apoptosis, differentiation	[124]
MRP1/ABCC1	lung carcinoma	multidrug resistance	decreased survival	[125]
NF90	tumor	DNA repair	DNA damage repair inhibition	[52]

**Table 4:** miR-7 target genes/proteins involved in differentiation and metabolic processes.

Gene/Protein	CELL TYPE	Pathway	Function	Reference
TLR4	brain	inflammation	downregulation of inflammation	[126]
FAM177A	macrophages	inflammation	inhibition of cytokine production	[127]
NLRP3 Nod like receptor	brain	inflammation	downregulation of inflammation	[128]
TET2	hematopoietic malignancies	chromatin modification		[58]
SETDB1 SETD8	pancreas	chromatin regulation		[56,57]
PAX6	brain lung colon pancreas embryonic stem cells		differentiation	[5,129]
Gli3	brain bladder cancer	hedgehog	differentiation	[130,131]
FGFR4	liver	protection from injury	stem cell proliferation	[132]
HoxD family	brain		differentiation	[133]
TCF4 and TCF12	brain	wnt pathway	differentiation	[134]
TCF7L2	brain	wnt pathway		[134]
SHANK3	brain		differentiation	[135]
ihog Hedgehog receptor	drosophila eye	hedgehog pathway	differentiation	[136]
CRY2	osteoblast	CLOCK/BMAL/p300 pathway	differentiation	[137]
Yorkie	drosophila wings	Hippo pathway	organ size	[138]
G protein signalling 5 RGSS	eye	signaling		[139]
PA28 gamma	lung carcinoma	proteasome	inhibition of protein degradation	[140]
insulin receptor INSR insulin receptor substrate 2 IRS-2 insulin-degrading enzyme IDE	brain	regulation of glucose metabolism	insulin sensitivity	[141]
TfR1 transferrin receptor 1	pancreatic and colon cells	iron transport and storage	iron transport and storage	[142]
beta arrestin 1	pancreatic beta cells	regulation of insulin secretion	metabolism	[143]
Sepp1b selenoprotein P	brain	synaptic function		[144]
Prostaglandin F2 receptor negative regulator PTGFR Golgi glycoprotein 1	pituitary gland	hormone regulation	gonad development	[145,146]
OGT, O-GlcNAcyl Transferase	lung cancer	O-GlcNAcylation	metabolic reprogramming migration	[147]
CAMK2D calponin	smooth muscle cell	calcification	vascular calcification in pulmonary hypertension	[148]
enolase ENO2	nasopharyngeal carcinoma	glycolysis	metabolism radioresistance	[149]
Lactat dehydrogenase A	gastric cancer	glycolysis	metabolism	[150]
Raf1	pituitary gland	production of prolactin	development	[151]
KLF4	myoblasts	differentiation and proliferation	inhibition of differentiation and proliferation	[152]
Follicle stimulating hormone FSH	pituitary gland	metabolism	inhibition of production	[48]
alpha-Synuclein	brain; Parkinson disease	neuron function and survival	inhibition of production	[153]

## Data Availability

Not applicable.
